# Supplementary material for: Circular photogalvanic spectroscopy of Rashba splitting in 2D hybrid organic–inorganic perovskite multiple quantum wells
Source: Nat Commun. 2020 Jan 16;11:323. doi: 10.1038/s41467-019-14073-6 (PMC6965620; doi:10.1038/s41467-019-14073-6)
Supplement: Supplementary file 1 — Supplementary Information [file 41467_2019_14073_MOESM1_ESM.pdf]

Supplementary Materials

Circular photogalvanic spectroscopy of Rashba splitting in 2D hybrid organic-inorganic perovskite multiple quantum wells

Xiaojie Liu[1], Ashish Chanana[1,2], Uyen Huynh[1], Fei Xue[3,4], Paul Haney[3], Steve Blair[2], Xiaomei Jiang[5*] and Z. V. Vardeny[1*]

[1]: Department of Physics & Astronomy, University of Utah, Salt Lake City, UT 84112, USA

[2]: Department of Electrical Engineering, University of Utah, Salt Lake City, UT 84112, USA

[3]: Center for Nanoscale Science and Technology, National Institute of Standards and Technology, Gaithersburg, MD 20899, USA.

[4]: Institute for Research in Electronics and Applied Physics & Maryland Nanocenter, University of Maryland, College Park, MD 20742, USA

[5]: Department of Physics, University of South Florida, Tampa, FL 33620, USA

1. **Supplementary Methods (SM)**

SM1. Samples preparation

PbI_2, R-NH_3I (where R is $C_6H_5C_2H_4$), N,N'-dimethylformamide (DMF), g-butyrolactone (GBL), and dichloromethane (DCM) were purchased from Sigma-Aldrich Corporation. All the materials were used as received without further purification.

All samples were fabricated in a nitrogen-filled glove box with oxygen and moisture levels of <1 part per million. We have grown the 2D hybrid perovskite (2D-PEPI) single crystals on cleaned quartz substrates using the Anti-solvent Vapor-assisted Crystallization (AVC) method as in ref. [1]. The 2D-PEPI crystals were used for the following measurements: photoluminescence (PL) spectrum, terahertz emission spectroscopy, XRD and SEM microscopy. For continuous-wave (CW) PGE measurement, two 70 nm thick gold electrodes were deposited onto the crystal by e-beam evaporation through a shadow mask in a glove-box-integrated vacuum deposition chamber (Angstrom Engineering), which had a base pressure of 3×10^{-8} torr ($\approx 4 \times 10^{-6}$ Pa). The gap between electrodes was 0.5 mm. **Supplementary Figure 1 (B)** is a photo of the device.

For 2D-PEPI film we mixed R-NH_3I and PbI_2 in a 2:1 molar ratio in DMF solvent to form a solution with a concentration of 0.2 mol/ml. This solution was stirred overnight at 60 ℃ on a hotplate before using. Subsequently the solution was spin-coated on an oxygen plasma–pretreated glass substrate at 314 rad/s and 90 s to form 100 nm thick film; the obtained film was subsequently annealed at 100 °C for 30 min. We used this film for the optical density measurement.

SM2. Continuous wave (CW) PGE measurements

CW diode lasers that operate at wavelengths of 405 nm, 447 nm, 486 nm, 520 nm and 532 nm, respectively were used to excite the 2D-PEPI single crystal between the two gold electrodes of the device. The laser beam with a diameter of 0.25 mm to 0.45 mm was focused exactly at the center between the two electrodes to minimize the effects caused by electrode asymmetry. In these measurements, the laser power was reduced to 45 μW, with a diameter of 0.35 mm (**Supplementary Figure 1 (A)**), so that the light intensity was 31 mW/cm^2. For measuring the CPGE action spectrum, we also used as a pump excitation an incandescent light source from a xenon lamp, which was dispersed through a monochromator. Roughly 25 % of the light beam was focused on the active area of the device, with an area 0.5 mm x 0.75 mm (**Supplementary Figure 1 (C)**), with an intensity of 8.0 mW/cm^2. **Supplementary Table 1** lists the comparison between actual power and intensity on the device, and **Supplementary Figure 1 (A-C)** shows photos of the device and beam sizes of these two cases. A comparison of PGE current is shown in **Supplementary Figure 1 (D,E)**. A quarter (λ/4) waveplate (QWP) and a half (λ/2) waveplate (HWP or polarizer) were used to modify the polarization property of the exciting light before the sample. In both cases the incident light is p-polarized. The light beam intensity was modulated at a frequency of 310 Hz, and the photocurrent was measured using a lock-in amplifier. Due to the very low intensity of Xenon lamp, we use full-slit width of the monochrometer to ensure the needed intensity for measuable signal. **Supplementary Figure 1 (F)** shows a lineshape of the xeon light after the monochrometer at wavelength of 510nm. This reduced spectral resolution has resulted in ±22-24meV uncertainty in the energy range used in **Supplementary Figure 2(B)**.

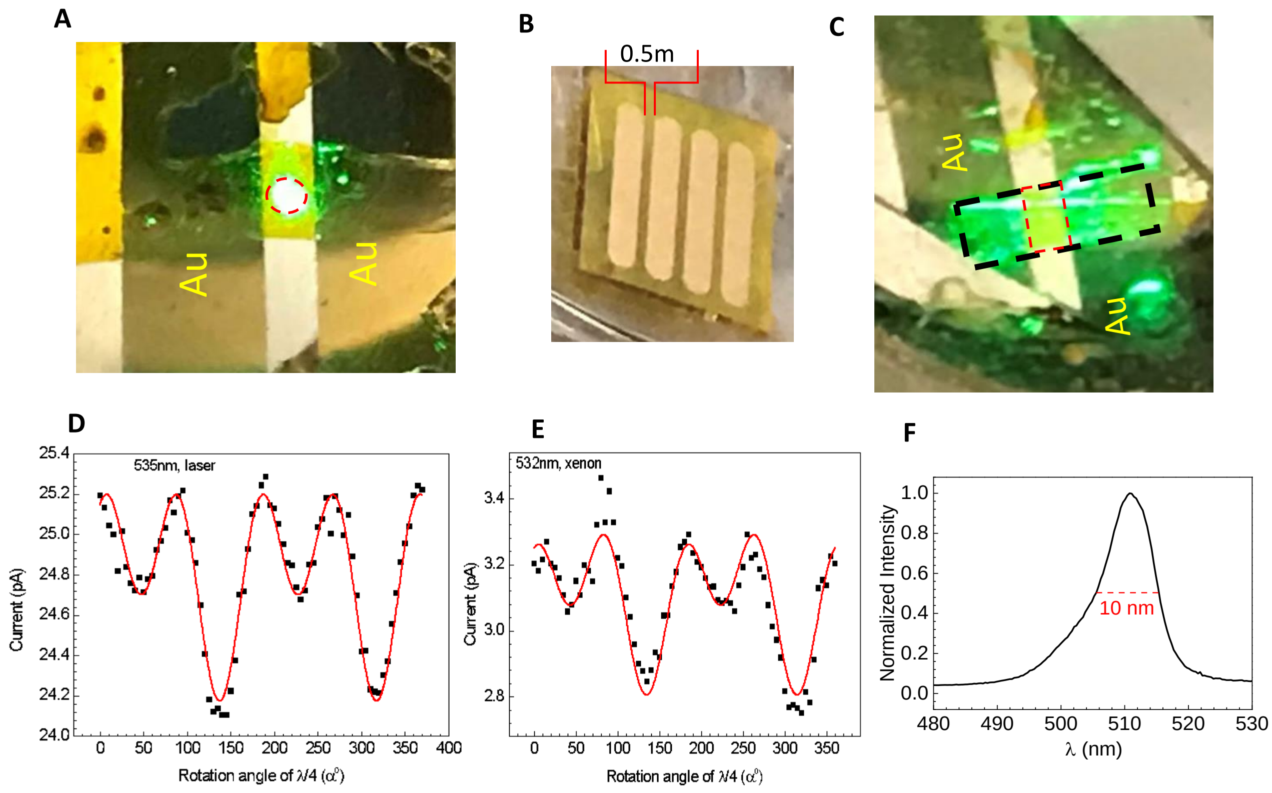

Supplementary Figure 1. (**A**) A photo of the PGE device under laser illumination at 535 nm. The red dashed line outlines the beam size (disk with diameter 0.25 mm). (**B**) Picture of a thin film device showing the device geometry. (**C**) The device under xenon light illumination. The black dashed line shows the full beam size, which is roughly 4x of the beam (0.5 mm x 0.75 mm) on the device (red dashed line). The wide dark bar is the Au electrode, and the narrow bright bar is the crystal. (**D**)-(**E**) PGE current in 2D-PEPI crystal vs. quarter- wave plate angle, α (at $\theta=35^0$), with (**D**) CW excitation of laser, 535 nm, power = 45 μW; and (**E**) xenon lamp, 530 nm, power = 30 μW. The redline is fitting using equ. (1) in main text. The extracted parameters are listed in **Supplementary Table 2**. (**F**) The profile of Xe lamp after the monochromator at 510nm. The full width at half maximum (FWHM) is about 10 nm. So the wavelength error bar for CPGE measurement is ±5nm. The corresponding energy error bar at this wavelength is about ±24meV.

Supplementary Table 1. List of experimental conditions under laser and xenon illuminations, respectively.

Light source	Beam size (mm^2)	P (μW)	Percentage on device	Actual power $\tilde{P}$(μW)	Intensity (W/cm^2)
Laser	0.096	45	100%	45	$4.7x10^{-2}$
Xenon lamp	0.375	30	25%	7.5	$2x10^{-3}$

Supplementary Table 2. List of all fitting parameters (C1,C2,L1,L2 and D) of **Fig. S1(D,E)** using equ. (1) in main text.

λ(nm)	C1	C2	L1	L2	C1/L2	D
535nm, laser	0.26±0.01	-0.02±0.01	0.06±0.01	0.36±0.01	0.72	24.8±0.05
532nm, xenon	0.13±0.01	-0.01±0.01	-0.01±0.01	0.16±0.01	0.85	3.1±0.05

As can be seen, C1 and L2 are about one order of magnitude larger than C2 and L1, respectively. Under laser illumination, D is about 8 times larger and the actual power 6 time larger as those obtained using the xenon lamp. This is probably due to larger photothermal effect from smaller laser beam [2]. Furthermore, C1(L2) with laser illumination is roughly 2x as much as with xenon. Nevertheless, the ratio between CPGE (C1) and LPGE (L2) does not change too much (within 20 %) in these two cases. These test results show that extra care must be taken to make sure any comparisons are conducted under same experimental conditions.

SM3. $\lambda/4$ waveplate (QWP) effect on the CPGE results

There was a recent report on possible artifacts from the rotation of $\lambda/4$ waveplate (QWP). A summary of this report is as follows: (1) intensity variation through the QWP; (2) photothermal current modulated by the QWP [2]. While the former is mainly due to nonuniformity of the waveplate and has period of 2π, the latter can bring in both sine and cosine terms of 2α and 4α, possibly complicating the real CPGE and LPGE terms. These artifacts cause particularly serious problems in the work of ref. [2] because the material used was a semimetal, and the QWP was at mid-infrared (CdSe) which has larger nonuniformity over the QWP area than the quartz waveplate we have used. We have conducted thorough investigations on our system and concluded that these artifacts are negligible, and do not cause alternation of the real experimental data. Details of our investigation are presented:

(1) Influence of the light intensity variation after the $\lambda/4$ waveplate on the C, L, and D parameters

We measured the power of excitation light vs the rotation angle of the $\lambda/4$ waveplate at three different wavelengths, 480 nm (IB), 530 nm ($EX_{\sigma+}$) and 535 nm, as shown in **Supplementary Figure 2 (A).** The power of the excitation light was measured in the same setup for CW PGE current measurement by replacing the device with a depolarizer and a silicon detector. To show that the variation of the excitation light power has little effect in the PGE(α) spectra, we divided the PGE current with the normalized power of the excitation light. The original C1/D at 480 nm, 530 nm and 535 nm were 1.80 %, 1.72 % and 1.05 %, respectively. Whereas the corrected C1/D at 480 nm, 530 nm and 535 nm were 1.73 %, 1.68 % and 1.08 %, respectively. **Supplementary Figure 2 (B-D)** show the change on PGE (α) spectra after the correction, and **Supplementary Table 3** lists details of $\lambda/4$ waveplate effect on other parameters. As can be seen, the intensity variation from the $\lambda/4$ waveplate affects C1 about 2 % to 4%, and L2 4 % to 7%. Therefore, we conclude that the variation of the $\lambda/4$ wave-plate transmission has little effect on the PGE(α) spectra. The larger fluctuation in C2 and L1 are due to the much smaller magnitude of these coefficients. In any case C2 and L1 are not important for the discussion since the CPGE and LPGE are dominated by C1 and L2, respectively.

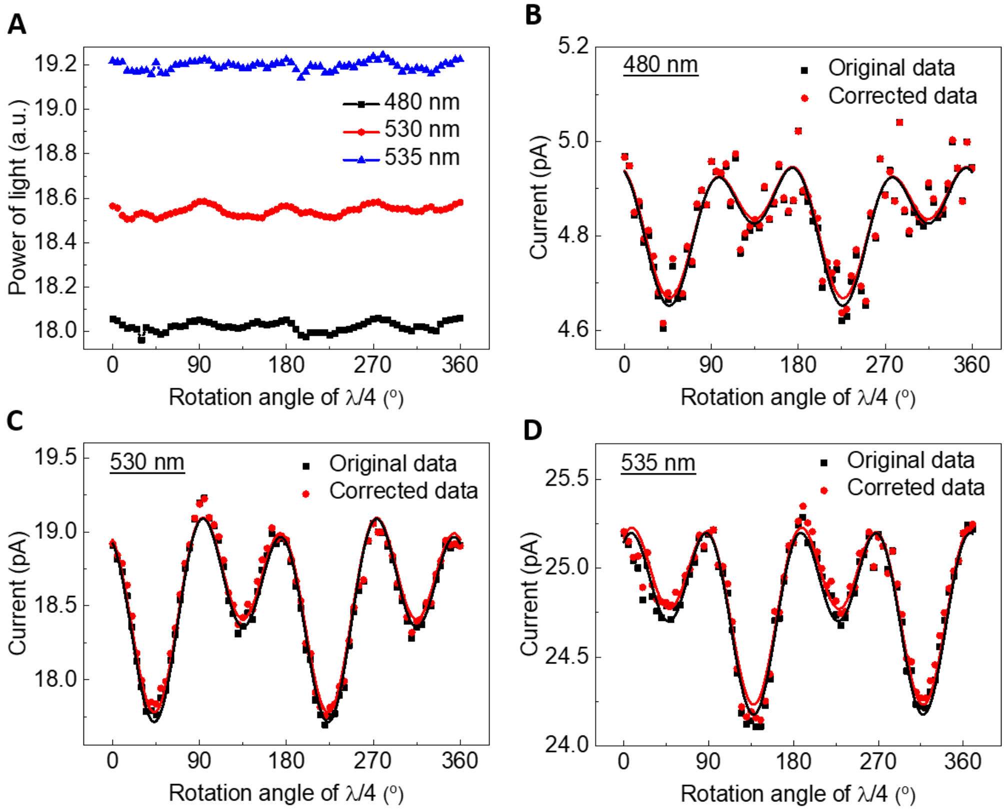

Supplementary Figure 2. (**A**) The power of the excitation light vs. the rotation angle α of the λ/4 waveplate at three different wavelengths, 480 nm (black), 530 nm (red) and 535 nm (blue). (B)-(D) The original PGE (α) current (black dot) and the corrected PGE (α) current (red dot) normalized by the excitation power at excitation 480 nm (**B**), 530 nm (**C**) and 535 nm (**D**). The black and red lines in the panels are fittings using the equ (1) in the main text. All the data are measured at the same conditions as those in **Fig. 2B** in the main text.

Supplementary Table 3. List of the λ/4 waveplate effect on the fitting parameters (C1,C2,L1,L2).

λ (nm)	**ΔC1/C1 (%)**	ΔC2/C2 (%)	ΔL1/L1 (%)	**ΔL2/L2 (%)**
535	**3.0**	40	5.5	**6.9**
530	**1.9**	1.0	1.0	**4.5**
480	**3.8**	60	60	**6.1**

(2) Misalignment (α_0) between the λ/4 waveplate fast axis and light excitation polarization direction

This unintentional and inevitable experimental error, α_0 was estimated to be less than a few degrees, mostly 1 degree to 5 degree. This does not place much difference in the C's and L's values.

(3) Photothermal current /Seebeck effect

According to ref. [2], photothermal current has both sin(2α) and cos(2α) (for the CPGE terms) and sin(4α) and cos(4α) (for the LPGE terms). However, this effect is minimal in our samples because:

1) Low free carrier density (n $\approx 10^{13}$ cm^{-3}) and low carrier mobility (μ=(0.1 to 1) $cm^2/V\cdot s$, comparing with the reference semimetal sample (n≈ (10^{18} to 10^{19}) cm^{-3}; μ=(10^4 to 10^5) $cm^2/V\cdot s$).

2) Very thick sample with average thickness of (8 to 10) μm, more than 20x the penetration depth, therefore the difference in absorption coefficient along the fast and slow axis of λ/4 waveplate does not matter *since all photons are absorbed in any case*.
3) The only possible non-zero term that might have small contribution to the PGE current is the cos(2α) term, estimated to be about 0.3 % of the DC background current. Our C1 (for sin(2α) is between 1 % to 5 % of the total DC background current (D). Since C2 (for cos(2α)) is 1 order of magnitude smaller than C1, it is possible that photothermal current makes some contribution in the cos(2α) term.
4) Very low power of xenon lamp (<30 μW) and large beam size (cover the whole device area) further minimize the Seebeck effect.

In conclusion, we believe that the PGE current that we measured is basically free from known artifacts.

SM4. Photoconductivity action spectrum

In this measurement, the incandescent light from a Xe lamp, which was dispersed through a monochromator, was used to excite the same device used in the CPGE measurement. We also measured the conductivity by sweeping the voltage applied to the device with a Keithley 238 multimeter [3]. The voltage was swept in a symmetrical way (0 V to -5 V, +5V to -5 V, -5V to 0 V). The photoconductivity was then subtracted by linearly fitting the I-V curve from +5 V to -5 V. This procedure was adopted from ref. [4].

SM5. Action spectra of the CPGE (C1), LPGE (L2) and DC offset (D)

Supplementary Figure 3 (**A-C**) shows the action spectra of circular photogalvanic effect (CPGE), linear photogalvanic effect (LPGE) and the DC offset (D) at resonant excitation of exciton with various azimuthal angles ϕ'. Please note that there is an angle ψ between the azimuthal angle in measurement (ϕ') and that in the theoretical calculation (ϕ) (see the experiment setup in **Fig. 1C**). As can be seen in **Supplementary Figure 3**, both CPGE and LPGE depend on ϕ', and there are polarity change of the photocurrent for both LPGE and CPGE. However, there's minimal dependence of D on ϕ', due to the slight intensity variation at different ϕ' angle. **Supplementary Figure 3 (D)** also shows that CPGE (C1) is not the first derivative of absorption(d(OD)/dE), for both exciton and interband excitations. **Supplementary Figure 4 (A)** compares the D internal quantum efficiency (IQE) spectrum with that of the photoconductivity (PC) IQE spectrum, both are normalized. D-IQE spectrum is very similar to the PC-IQE spectrum measured at -5V bias, inferring that there is a weak electric field within the device although the applied voltage is zero. We speculate this weak field comes from the photothermal effect (proportional to absorption) and photovoltaic effect (from slight asymmetry among the two electrodes) [2,4]. The two IQE spectra have nearly identical onset at both exciton and IB transitions, where the absorption has minima. Based on the comparison between PC current @ (-5V) and the DC offset current D, we estimate the internal electric field E_{PGE} is about 2 orders of magnitude smaller than the electric field E_{PC}=5(V)/10μm (in the device used for PC measurement). Since the two crystals have similar thickness (~10μm), $E_{PGE}=10^{-2}E_{PC}=10^{-2}(5(V)/10\mu m)) = 5x10^4$ V/m=$5x10^2$V/cm, which is 3 orders of magnitude smaller than a typical electric field in electroabsorption measurement [5]. As another comparison, we took a look at the electric field inside a typical solar cell made with similar RP 2D perovskite [6]. At the exciton energy (2.5eV), the external quantum efficiency is about 5%. The built-in electric field in their device is estimated as:

$$E_{built-in} = \frac{\Delta V_{buit-in}}{d} = \frac{0.5V}{200nm} = 2.5 \times 10^4 \, {}^{V}/_{cm} >> 500 \, {}^{V}/_{cm} \; in\ our\ PGE\ device$$

Therefore we expect the internal electric field at zero bias in our device used for PGE measurements is too weak to dissociate the excitons.

It is interesting to see that the IQE for exciton and IB carriers are almost the same in the PC action spectrum; however, more than doubled in D action spectrum. Due to the much stronger electric field (> 2 orders) in PC measurement, both exciton dissociation and free carriers (electrons and holes) separation may be equally efficient. Based on the coincidence of CPGE peak with the lower energy exciton (EX1) at 2.3 eV, we conjecture that the exciton might dissociate via the edge states, or other native defect sites [6].

Supplementary Figure 4(B) shows a comparison between the PC-IQE and absorption spectra at room temperature and low temperature (10K). It is seen that the redshift of PC with respect to absorption is much less at T=10K, when the localized/trap states at the absorption edge become unavailable (or frozen).

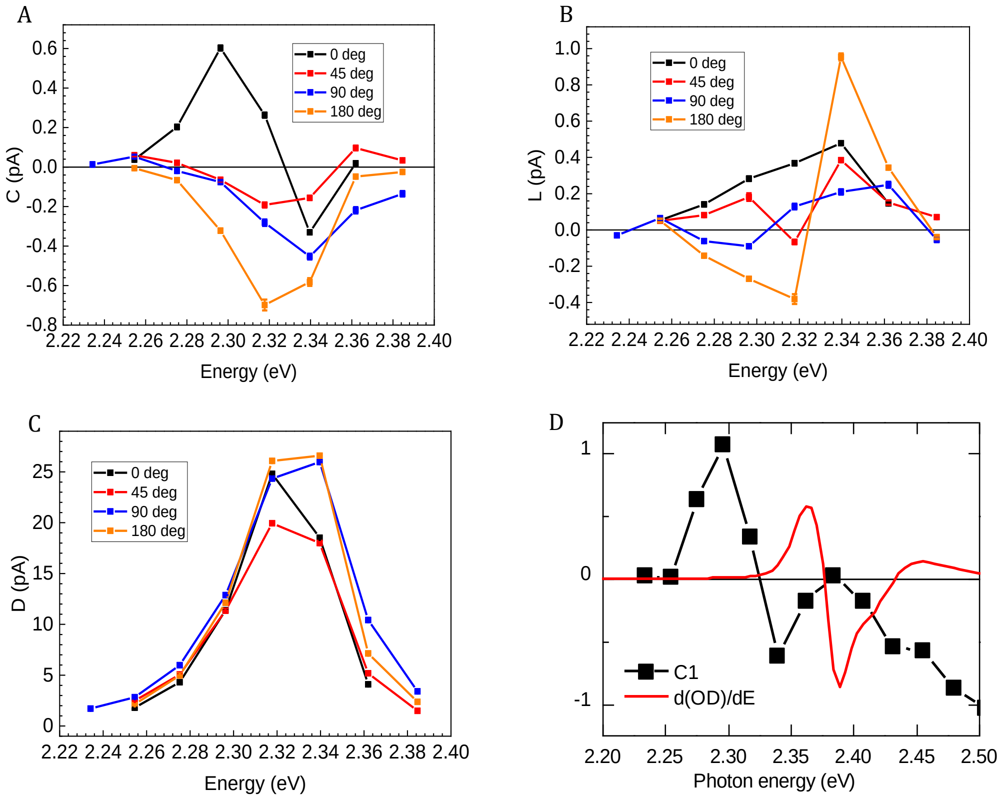

Supplementary Figure 3. Action spectrum of (**A**) C1, (**B**) L2, (**C**) D at resonant excitation with the exciton band at θ=30^0 with various ϕ' angles, as shown in each figure. (**D**) shows the comparison between (C1) at ϕ=0^0 (black symbol) with the first derivative of the absorption spectrum (red line). We conclude from this comparison that C1 spectrum is not due to the derivative of the absorption spectrum.

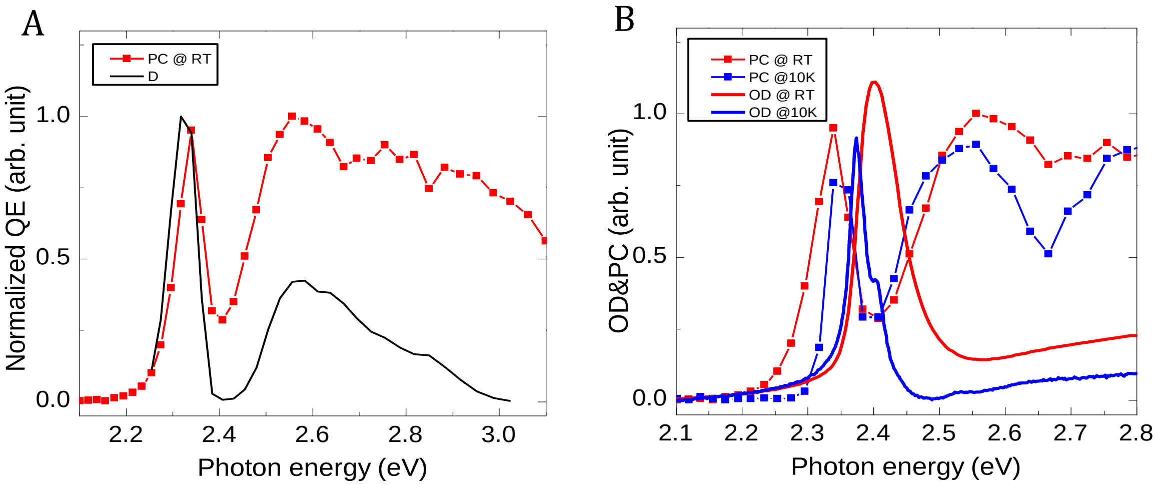

Supplementary Figure 4. (**A**) Normalized internal quantum efficiency (QE) action spectrum of photoconductivity (PC) (symbol) and DC offset D (line); PC measurement was taken with a reverse bias of 5 volt; (**B**) Comparison of the absorption at room temperature (RT, red line) and T=10 K (blue line); PC-IQE at room temperature (RT, red symbol) and T=10 K (blue symbol). At low temperature, the absorption red-shifts while PC-IQE blue-shifts.

SM6. SEM and XRD characterizations of the 2D-PEPI crystals

The 2D-PEPI crystals were characterized using a scanning electron microscope (SEM), FEI Helios Quanta, at low vacuum of 4.8 mTorr (≈0.064 Pa), to avoid charging effects on sample and substrate. Images were taken at different magnification showing uniform growth of ≈ 3 mm size crystals, where higher magnification images at the edge of the crystal show layered structure highlighting the 2D layered structure of the crystals. **Supplementary Figure 5** shows SEM images at 57X and 500 X. The film thickness was characterized using a profilometer, Tencor P 10. The thickness was measured along across the length of crystals showing a fairly smooth morphology with a nominal crystal thickness was found to be 8 μm to13 μm thick, consistent with that in ref [1].

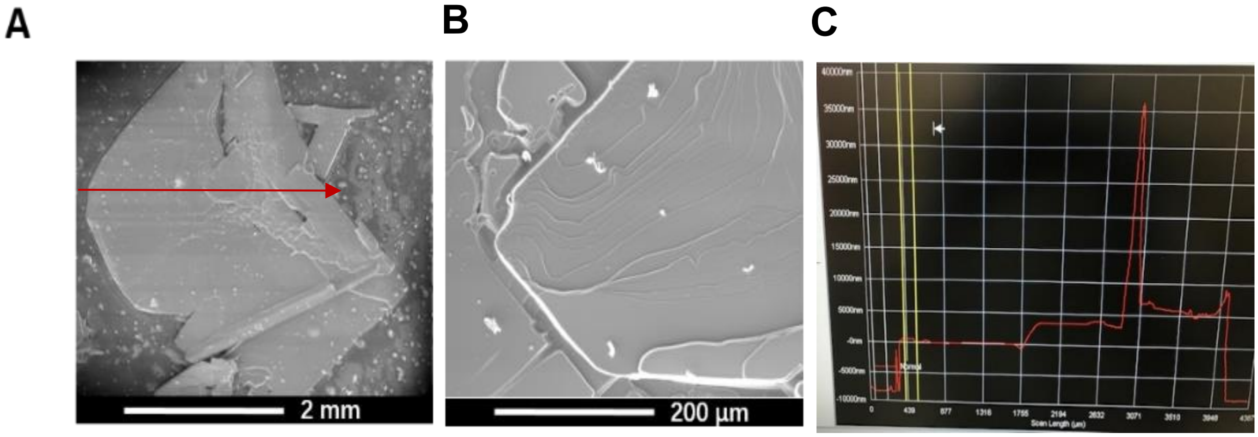

Supplementary Figure 5. Scanning electron micrograph (SEM) images of 2D-PEPI crystals. **(A)**. The crystal shown is about 2 mm x 4mm. **(B)**. Zoom-in image shows the layered structure, with the stacking direction normal to

the quartz substrate. **(C)**. The height profile measured along the redline in A. The spike was from an anomaly also shown as a white dot in A.

Finally (out-of-plane) x-ray diffraction pattern was measured using Bruker D2 Phaser. The 2-theta scan shows highly crystalline growth along the c-axis, as shown in **Supplementary Figure 6**. The calculated inter-layer distance is c = 3.25 nm, consistent with earlier reports [7, 8]. SEM images combined with XRD spectrum points to relatively large single crystal [1].

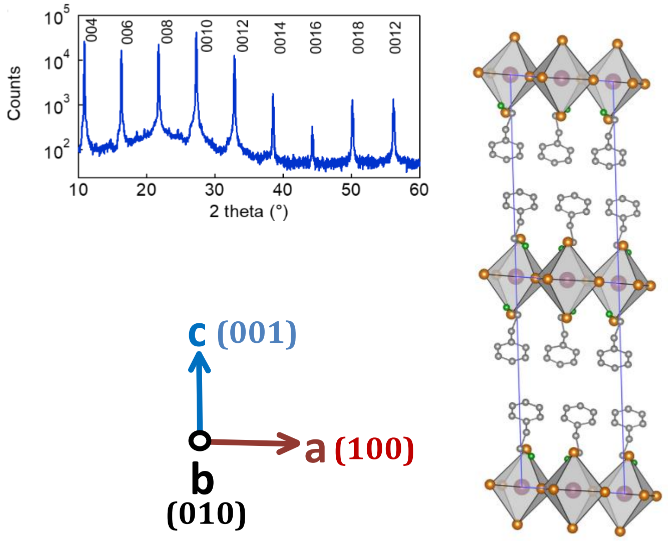

Supplementary Figure 6. X-ray diffraction pattern (XRD) of 2D-PEPI crystal grown on quartz substrate (upper left panel). The X-ray source is Cu-K_α line (λ=0.154 nm). The right panel shows the side view of the crystal structure with $[PbI_6]^{4-}$ layers parallel to the quartz substrate.

SM7. AFM measurement of the 2D-PEPI single crystal

The 2D-PEPI single crystal morphology and roughness were measured using BrukerDimesion Icon atomic force microscope (AFM) utilizing the scan-assist tapping mode. A region of 25 μm x 25 μm was scanned with lateral resolution of 12.2 nm and height resolution of ≈2 nm using AFM tip having 2 nm nominal diameter. The data was analyzed using Bruker Nano scope Analysis software where prior to image processing, a linear flattening was applied to account of tilts and low frequency noise. The average roughness was computed using RMS over the complete scan.

Supplementary Figure 7 shows the topography of two crystals that we measured. Crystal 1 shows a terrace like structure over multiple layers corresponding to 2D nature of the PEPI crystals. The larger roughness was a result of scanning across multiple layers (4 layers), so the average roughness is about 3.34 nm to 4.675 nm. The scanning region of Crystal 2 was on single layer which shows its smooth surface with roughness of 3.82 nm. The observed step height is also consistent with the c = 3.25 nm estimated from XRD (see **Supplementary Figure 6** and discussion).

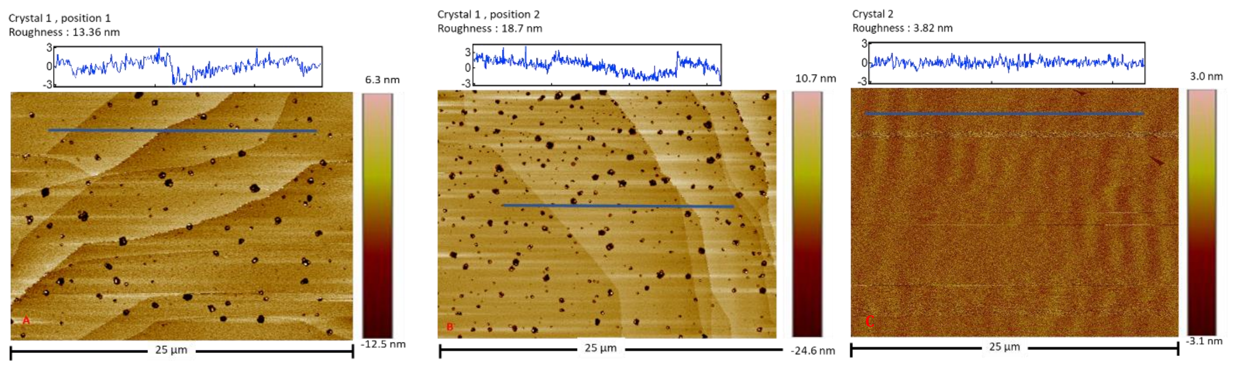

Supplementary Figure 7. AFM topography of two different 2D-PEPI crystals. The dark spots shown in A&B are tiny pin holes (smaller than sub-micrometer).

SM8. Other optical characterizations

All optical measurements were done at room temperature in air. The absorption (or optical density) was measured using a UV/Vis spectrometer (Olis). For the photoluminescence (PL) measurement, a 2D-PEPI single crystal was excited using a 30 mW CW laser at 486 nm. We used two different setup configurations, respectively to measure the PL emission spectrum from the flat side of the crystal (surface-PL) and PL emission from the edge of the crystal (edge-PL) (see **Supplementary Figure 8, inset**). In both cases, the PL spectrum was recorded by an Ocean Optics USB4000 spectrometer via an optical fiber. The two different PL spectra are shown in **Supplementary Figure 8**. Both spectra show a dominant contribution from excitons. The edge PL band is red-shifted with respect to the surface-PL band by about 60 meV, similar to previous reports in other 2D lead perovskites crystals [6,9]. The edge effect indicates the existence of layer edge defect states which were shown to facilitate excitons dissociation into long-lived free carriers [6].

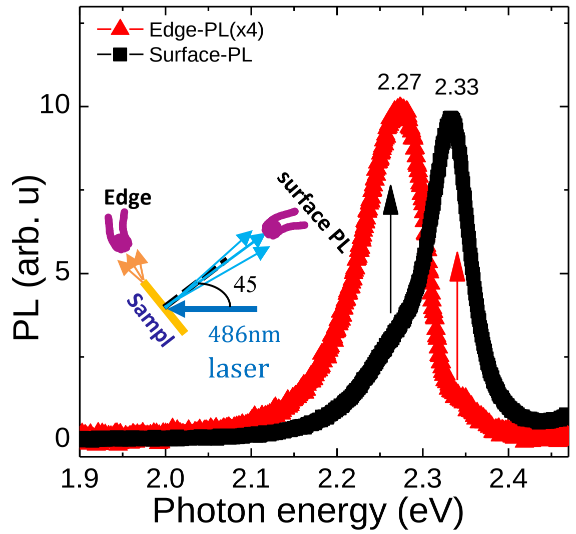

Supplementary Figure 8. Room temperature photoluminescence (PL) spectra of 2D-PEPI crystal collected from the front (surface-PL, black symbol) and the edge of the crystal (edge-PL, red symbol). The excitation was a CW 486 nm diode laser with power of 30 mW, and the spectra were recorded by a commercial Ocean Optics USB4000 spectrometer.

The surface-PL band peaks at 2.33eV namely EX2 in **Fig. 1B**, with low energy shoulder at the same position as the edge-PL peak (2.27eV). On the other hand, the edge-PL spectrum contains a small high

energy shoulder that coincides with the surface-PL band. The mutual inclusion of these two PL band in the two measurement geometries indicates the co-existence of free exciton and edge state exciton in our 2D-PEPI crystal.

SM9. Terahertz emission measurements

***Set-up*.** Terahertz emission from 2D-PEPI crystals was measured by an electro-optic sampling technique using standard time-domain spectroscopy configuration. The samples were excited by 0.25 μJ pulses at 405 nm generated using type-I BBO crystal pumped with 810 nm pulses from Ti-Sapphire regen-amplified laser system at 1 KHz repletion rate. 2D-PEPI crystals on Quartz substrate were excited from the quartz side. The emitted terahertz radiation due to photo-excited carriers was collected by 2 parabolic mirrors and focused on to 0.5 mm thick electro-optic ZnTe <110> crystal. The terahertz field pulse signal was measured as the change in polarization of the probe beam induced as a result of electro-optic sampling technique, as measured by a Wollaston prism and a set of balanced silicon detectors using lock-in technique. We note that the measured bandwidth of the emitted signal detection technique is limited by the detection crystal. To measure the polarization dependent terahertz field, the sample was mounted on a rotation stage and the excitation beam was modulated using λ/2 and λ/4 plate. In addition, a wiregrid polarizer was placed in the collimated beam path between the two parabolic mirrors, to allow detection of polarization emitted terahertz field. A schematic diagram of the experimental setup is shown in **Supplementary Figure 9**.

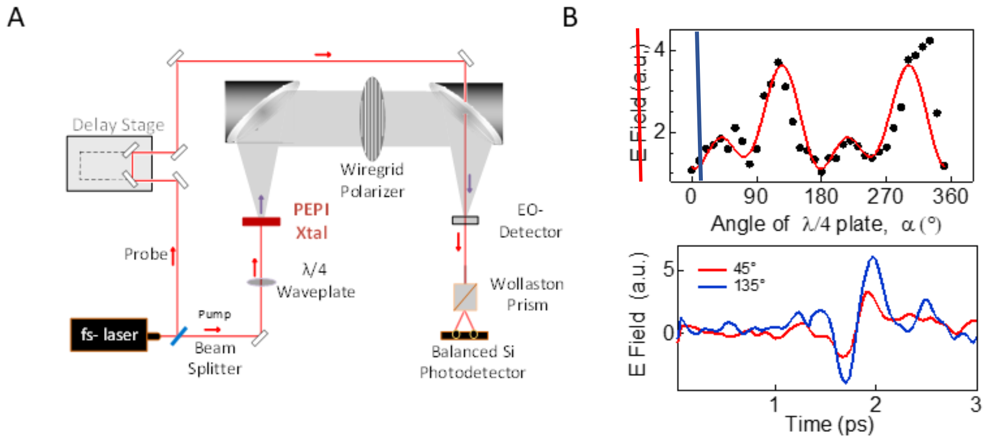

Supplementary Figure 9. (**A**). Schematic representing the time domain terahertz emission setup. (**B**). upper panel: THz emission field due to ultrafast photogalvanic current in 2D-PEPI crystals measured along x' direction, as a function of the rotation angle, α, between the excitation pump polarization and the fast axis of a λ/4 waveplate; lower panel: time domain THz signals at the marked α angles (α=45^0 for RCP, and α=135^0 for LCP. The excitation is from 3.06 eV of the fs pulse laser at θ=45˚.

Simultaneous Terahertz emission measurements for $E_{x'}$ and $E_{y'}$:

Since terahertz measurements are non-contact measurements it allows us to measure ultrafast currents along the two orientations simultaneously. Here the wiregrid polarizer, the probe beam and detection electro-optic crystals were rotated by 90˚ to measure the field emission along the orthogonal polarization. The THz field emission $E_{x(y)}$ is related to ultrafast photocurrent $J_{x(y)}(t) \sim e^{-\frac{t}{\tau}}$ in a simple form:

$$E_{x(y)} \propto \frac{dJ_{x(y)}(t)}{dt} \propto |J_{x(y)}(t)| \quad (1)$$

Therefore we expect the same formulae for $J_{x(y)}$ would apply to $E_{x(y)}$. For the dependence on rotation angle α of the λ/4 plate, we can use a similar form as equ. (1) in the main text to fit the experimental data, other than a proportional coefficient with the unit of $[A \cdot m/S]$.

$$E_{x(y)} \propto L1_{x(y)} \sin(4\alpha) + L2_{x(y)} \cos(2\alpha) + C1_{x(y)} \sin(2\alpha) + C2_{x(y)} cos\,(2\alpha)\ + D \quad (2)$$

Furthermore, as shown in **Fig.1C**, there is an angle ψ between the measurement x' and the crystal a-axis (x-axis in **Fig. 1C**). So, we have the following equations for the measured $E_{x'(y')}$:

$$E_{x'} = E_x cos\,\psi + E_y\ sin\,\psi \quad (3)$$

$$E_{y'} = E_x \sin\psi + E_y\ cos\,\psi \quad (4)$$

We focus on the two dominant parameters, namely, *C1* for CPGE, and *L2* for LPGE. Details on derivation of *C1* and *L2* can be found in **Auxiliary Supplementary Materials**.

Based on Supplementary equ. (2)-(4), for $E_{x'}$, we get:

$$C1_{x\prime} = C1_x cos\,\psi + C1_y\ sin\,\psi \quad (5)$$

$$L2_{x\prime} = L2_x cos\,\psi + L2_y\ sin\,\psi \quad (6)$$

For $E_{y'}$, we have

$$C1_{y\prime} = C1_x sin\,\psi + C1_y\ cos\,\psi \quad (7)$$

$$L2_{y\prime} = L2_x sin\,\psi + L2_y\ cos\,\psi \quad (8)$$

With:

$$C1_x = sin\theta\ sin\,\phi\gamma_{xy} - cos\,\theta\gamma_{xz}\ (S9)$$

$$C1_y = -sin\,\theta cos\,\phi\gamma_{yx}\ \ (S10)$$

$$L2_x = -\frac{1}{4} sin\,(2\theta)\ cos\phi\chi_{xxz} + \frac{1}{8}(3 + cos\,(2\theta)\ )sin\,(2\phi)\ \chi_{xxy}\ \ (S11)$$

$$L2_y = -\frac{1}{4}\sin 2\theta \sin\phi\, \chi_{yyz} + \frac{1}{4}\left[\chi_{yxx}(\cos^2\theta\cos^2\phi - \sin^2\phi) + \chi_{yyy}(\cos^2\theta\sin^2\phi - \cos^2\phi)\right]\ \ (S12)$$

In this experiment, we use s-polarized excitation polarized along y' direction, so the plane of incidence is along x' direction therefore $\phi' = 0^0$, accordingly, $\phi=\phi'-\psi=0-\psi=-\psi$ (see **Fig. 1A**). For the case of normal incidence ($\theta=0^0$), Supplementary equ (9)-(12) can be simplified as:

$$C1_x = -\gamma_{xz}\cos\psi \quad (9')$$

$$C1_y = 0 \quad (10')$$

$$L2_x = -\frac{1}{2}\sin(2\psi) \quad (11')$$

$$L2_y = \frac{1}{4}\left(\chi_{yxx} - \chi_{yyy}\right)\cos(2\psi) \quad (12')$$

Supplementary equ.(5) –(8) then give:

$$C1_{x'} = -\gamma_{xz}\cos^2\psi \quad (5')$$

$$C1_{y'} = -\gamma_{xz}\sin\psi\cos\psi \quad (6')$$

$$L2_{x'} = -\frac{1}{2}\sin(2\psi)\cos\psi + \frac{1}{4}(\chi_{yxx} - \chi_{yyy})\cos(2\psi)\sin\psi \quad (7')$$

$$L2_{y'} = -\frac{1}{2}\sin(2\psi)\sin\psi + \frac{1}{4}(\chi_{yxx} - \chi_{yyy})\cos(2\psi)\cos\psi \quad (8')$$

From Supplementary equ. (5') and (6'), we can get a simple expression to determine the angle ψ as:

$$\psi = \tan^{-1}(\frac{C1_{y'}}{C1_{x'}}) \quad (13)$$

Supplementary Figure 10 shows the field emission profiles of two orientations (x'and y'). The red line is fitting using equ. (S2). **Supplementary Table 4** lists all fitting parameters. $\psi = (24\pm21)^0$ in this measurement. The big uncertainty is due to the not-so-satisfying fitting for $E_{y'}$ (goodness of fitting or adjusted-R^2 is only 0.74). As can be seen in **Supplementary Figure 11**, the error bars are generally bigger for *C1* than for *L2*, probably due to the small value of *C1* at normal incidence.

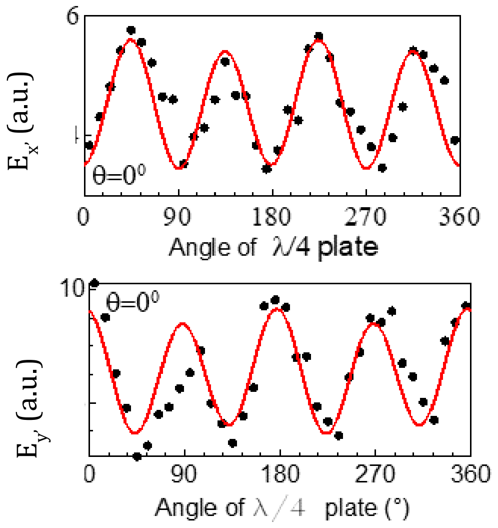

Supplementary Figure 10. Polarized terahertz emission due to ultrafast photogalvanic currents in 2D-PEPI crystals. $E_{x'}$ and $E_{y'}$ represent terahertz field emission along x' and y' direction respectively, as a variation of excitation pump polarization modulated by rotation of a λ/4 waveplate. The fields $E_{x'}$ and $E_{y'}$ were subsequently measured using probe beam and wire grid polarizer along the respective direction. The excitation is from 3.06 eV of the fs pulse laser at ϑ=0˚.

Supplementary Table. 4. The best fitting parameters for **Supplementary Figure 9** using Supplementary equ. (2).

Field direction	**C1**	C2	L1	**L2**	D
$E_{x'}$	**0.207±0.089**	-0.0881±0.149	0.0111±0.148	**-0.796±0.149**	4.58±0.106
$E_{y'}$	**-0.0928±0.124**	-0.195±0.131	-0.187±0.187	**0.981±0.199**	8.53±0.140

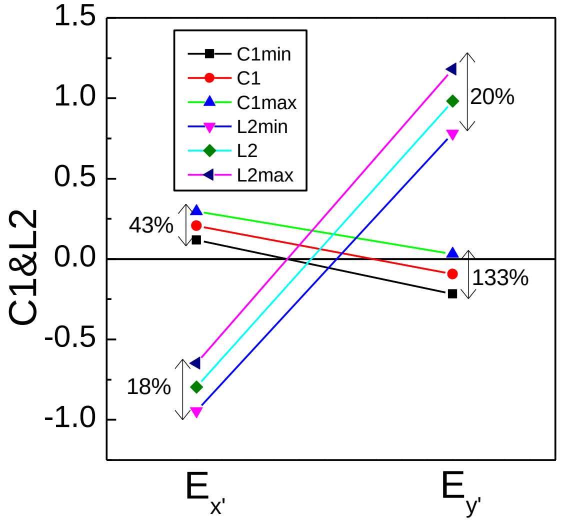

Supplementary Figure 11. Details of *C1* and *L2* in **Table S4**. with error range marked by the arrow (error bar, s.e.m.).

The analysis shows that we can use the simultaneous measurements of $E_{x'}$ and $E_{y'}$ to determine the unknown **angle** ψ for each crystal sample. It also can be seen that C1 in both directions are very close to zero, this indicates that the degree of in-plane inversion symmetry is much smaller within the plane (along y-direction or *b*-axis) than out-of-plane (along *z*-direction).

Terahertz emission of optical phonons

In order to distinguish the THz emission from optical phonons from that resulted from the ultrafast current in CPGE in 2D-PEPI as shown in **Supplementary Figure 9&10**, we measured the optical phonon modes using terahertz transmission through the crystal with respect to the reference substrate. **Supplementary Figure 12** below shows the THz emission from CPGE current (**Supplementary Figure 12 (A)**) and its Fourier transform spectrum (**Supplementary Figure 12 (B)**). For comparison, **Supplementary Figure 12 (C)** shows the measured absorption spectrum in the THz range through the PEPI crystal that shows the optical phonon modes at 0.78 THz and 1.6 THz, respectively. These phonons are probably associated with Pb-I-Pb rocking vibration and Pb-I stretching bonds as observed before [10]. It is worth pointing out that, while the THz emission from the ultrafast CPGE current is primarily at ~1THz (see **Supplementary Figure 9 (B) and Fig. 3B**), the frequency ranges at 0.8 THz and above 1.6 THz are suppressed. This is due to the fact that we measured terahertz emission in transmission mode through the crystal, where photo-absorption and emission happen from limited thickness of the crystal. The transmission of the emitted radiation though the thickness of 7-10 μm of the crystal would cause subsequent absorption of the signal to a significant amount at resonance with those phonon frequencies. Therefore, the measured THz radiation is peaked at 1 THz where the dip between the two phonons is observed. We thus conclude that the presence of emitted signal in **Supplementary Figure 9 (B) and Fig. 3B** at complementary frequencies to those of the phonons (**Supplementary Figure 12 (C)**).

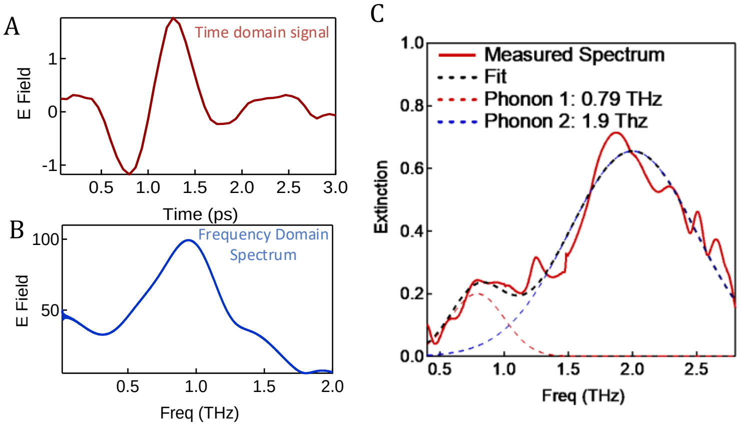

Supplementary Figure 12: (**A**). The transient THz emission and its Fourier transform spectrum (**B**) measured from PEPI crystal, compared to the crystal absorption spectrum measured in the THz range (**C**). The spectrum is decomposed into two phonon modes as indicated.

***Terahertz emission measurements upon varying the half wave plate (λ/2) angle*:**

We have also measured terahertz emission from 2D-PEPI upon varying the angle of λ/2 wave-plate, as shown in **Supplementary Figure 13,** left panel. The origin of the linear photogalvanic effect (LPGE) that results in terahertz emission could be attributed to two possible processes: *(i)* 'Optical rectification' as second-order non-linear process; and (ii) 'shift current' resulting from the displacement of the wave function center upon transitioning from valence to conduction band (as shown schematically in **Supplementary Figure 13**, right panel). Both mechanisms require non-centrosymmetric crystal symmetry which could arise of off-center positioning of lead ion, Pb^{2+}, in the in-plane PbI_6^{4-} octahedrons. Previous studies have identified a strong coupling between phonon modes and photoexcited states in 2D-PEPI, and other ferroelectric oxides such as $KTaO_3$ [11] exhibit a dynamic Jahn-Teller effect upon photoexcitation. The possibility of photoinduced changes in symmetry is reserved for future studies.

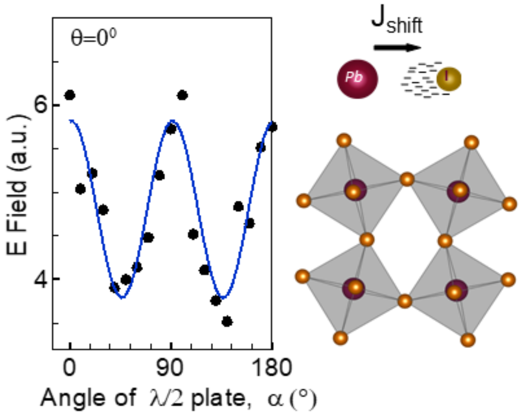

Supplementary Figure 13. Terahertz emission intensity as a function of the rotation angle of a λ/2 waveplate. The fitting is done using ~cos(4α) term. The schematic shown in the right panel represents photoinduced shift of charge to more electronegative I^- in Pb-I bond. This hypothesis relies on off-center Pb^{2+} in PbI_6^{-4} octahedron, as possible origin of non-zero shift current in 2D-PEPI crystal.

Excitation power variation as function of quarter waveplate (λ/4) angle:

To calibrate the uniformity of average power of 400 nm incident excitation pulses, we measured the average power using a polarization insensitive thermal power meter as a function of quarter wave plate angle. We calculated corresponding photon density, N, of excitation pulses using the relation $= \alpha \frac{\varepsilon_p}{E_{ph},A}$, where α is absorption coefficient, ε_p is the pulse energy, E_{ph} is the energy per photon at 400 nm and A is the excitation beam area. Given the defined parameters remains same, the photon density with rotation angle of λ/4 waveplate was found to be fairly constant, with random fluctuation less than 2 % (see **Supplementary Figure 14**). Therefore this small variation could be regarded as arbitrary and disregarded as the factor causing the observed trends of CPGE and LPGE.

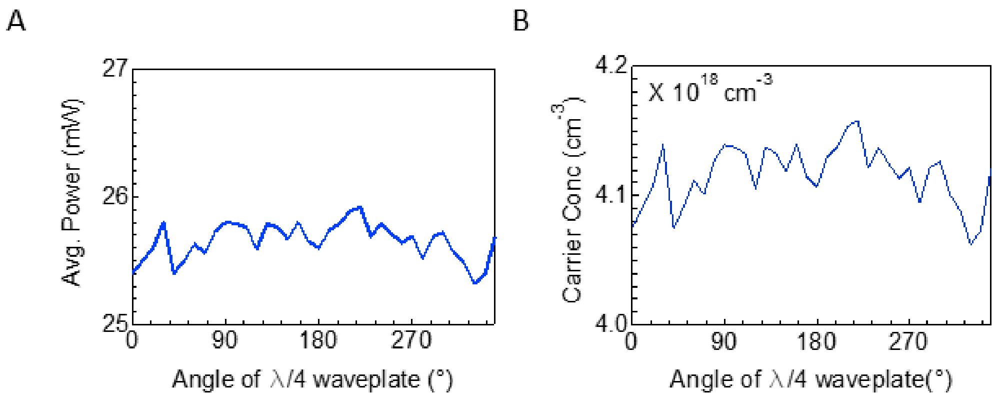

Supplementary Figure 14. (**A**) The power of excitation pulsed light vs. the rotation angle of the quarter-wave plate at 405 nm. **(B)** corresponding calculated carrier density as variation of angle of quarter wave plate. Overall 2 % change in carrier density was observed while no particular trend is present. This is estimated to have a minimal impact on trends observed in terahertz emission from 2D PEPI crystal.

SM10. Transient circularly polarized photoinduced absorption measurements

The transient polarization modulated photoinduced circular reflection/absorption (so called circular-PPA) apparatus is schematically shown in **Supplementary Figure 15**. It is a derivative of the well-known optical pump/probe spectroscopy. In regular pump/probe (or photoinduced absorption PIA) spectroscopy, the pump pulses that are modulated by an optical chopper or acoustic optic modulator (AOM) are absorbed by the sample and generate photoexcitations (excitons or electron and hole pairs); whereas the probe pulse is used to monitor the population in various states of these photoexcitations. Both pump and probe beams are aligned through various optical components in order to spatially and temporally overlap them on the sample. In this case the photoexcitations dynamics is measured by temporally delaying the probe pulses with respect to the pump pulses using a mechanically delayed stage.

Unlike the regular pump/probe spectroscopy, in our circular-PPA setup, only the polarization of the pump beam is modulated using a PhotoElastic Modulator (PEM) at 41 kHz, between left and right circular polarization. The probe beam is also circularly polarized for circular-PPA. In the present study, the pump and probe beams were split from the output of a Ti: Sapphire laser (Spectra-Physics) with pulse duration of 150 fs and 80 MHz repetition rate that can be turned from 730 nm to 810 nm. In addition, the pump beam was optically doubled to ~ 400 nm by a second harmonic generation crystal, whereas the probe beam was at ~ 530 nm from a combination of the idler beam with the fundamental at 800 nm. In order to

minimize the large scattering from the strong pump beam into the detector, we used a double modulation scheme in which in which the probe beam was also modulated by a mechanical chopper at 1.2 kHz. The pump beam with average intensity of 3 Wcm^{-2} and much weaker probe beam were focused onto a small area of the sample (either single crystal or thin film) having a spot size of ~100 μm in diameter. The probe beam reflected (or transmitted) intensity was measured with a silicon photodetector connected to the first lock-in amplifier that was externally synchronized with the chopper frequency. The second lock-in amplifier was externally synchronized with the PEM modulation frequency. This experimental set-up has a superior sensitivity for measuring the spin relaxation time than the transient Faraday rotation or regular polarized pump/probe technique.

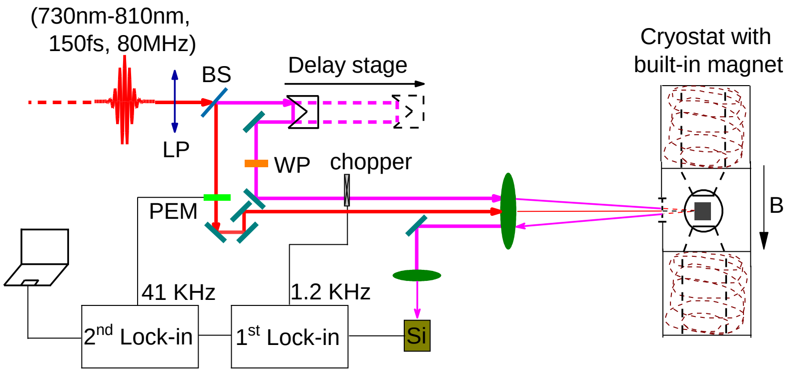

Supplementary Figure 15: Experimental apparatus for polarization modulated photoinduced circular reflection/absorption technique. PEM stands for a photoelastic modulator that changes the pump beam polarization between left and right circular polarization. WP is a quarter wavelength plate for the circular-PPR (or PPA); LP is a linear polarizer, and BS is a beam-splitter. Double lock-in technique was used to minimize the pump scattering into the photodetector.

Firstly, we measured a ‘circular polarization memory’ at the exciton level at room temperature using the transient polarized photoinduced absorption technique at 537 nm with 150 fs time resolution. In this method we set the pump beam polarization at a fix circular polarization, whereas the circular polarization of the probe beam was modulated between same circular polarization or opposite polarization to that of the pump beam. In this method only the difference between the same or opposite pump-probe circular polarizations is measured. Indeed, we found that there is ‘circular polarization memory’ for the exciton, in which the photoinduced absorption is larger when the pump-probe have same polarization than that of pump-probe with opposite polarization (see **Supplementary Figure 16**). This shows that the excitons are spin polarized upon absorption by a circular polarized pump and maintain the polarization even at room temperature.

Secondly, we measured the lifetime of this circular polarization memory. **Supplementary Figure 17** shows the transient circular PPA response of a 2D-PEPI crystal at room temperature. As can be seen, the spin relaxation process has two time constants (TC); a fast TC of 2.3 ps and a slower TC of 14.6 ps. The fast TC may be related to the exciton thermalization, whereas the slower TC is for the decay of thermalized excitons. The average spin relaxation time is about 4.5ps, meaning that the exciton and the resulting electron-hole pairs that follow exciton dissociation in 2D-PEPI lose their spin alignment within 4.5 ps, which is much longer than the momentum relaxation time in this material (~ 100 fs). This explains the lack of THz emission (ultrafast within tens of femtoseconds) from the photocurrent upon exciton

excitation with circularly polarized light. We therefore consider spin-galvanic effect (SGE) to be the mechanism for the photocurrent at exciton transition. Details are given in the main text.

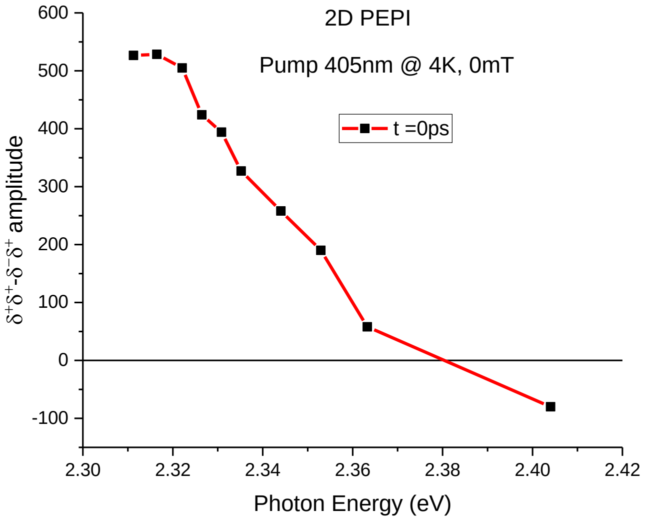

Supplementary Figure 16. Transient polarization modulated photoinduced circular absorption spectrum measured at time t=0 at 4K and zero magnetic field at 405 nm excitation. The signal at room temperature was too small for measuring the spectrum in this configuration. However, we were able to get the spin dynamics as shown in **Supplementary Figure 17**.

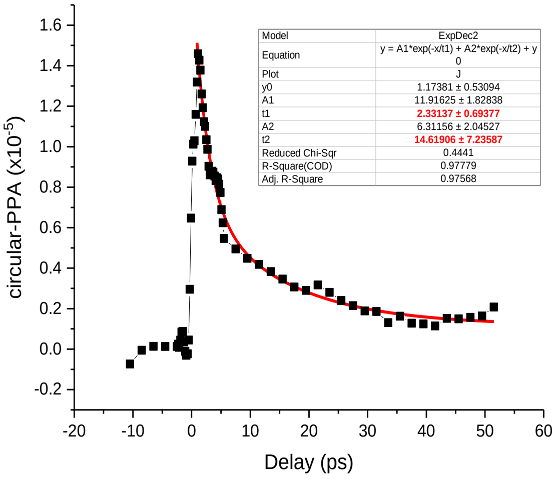

Supplementary Figure 17. Transient circularly polarized pump-probe absorption (PPA) of a 2D-PEPI crystal measured at room temperature using pump at 410 nm and probe at 537 nm, in resonance with the exciton transition. The line through the data points is a fit using a double exponential function with time constants of 2.3 ps and 14 ps, respectively, from which we obtain an average spin relaxation time of ~ 4.5 ps.

SM11. Theoretical calculation of symmetry tensors

Summary of the calculation: For the third order tensor, χ_{ijk} (symmetric among the 2nd and 3rd indices) related to the LPGE the first index indicates the current flow direction, and the 2nd and 3rd indexes stand for the optical field. For instance, χ_{xzx} corresponds to current flowing in the x-direction as a result of absorption of light of the form $\vec{E}(\hat{z}+\hat{x})$. Our model is capable of reproducing the experimental features, as seen in the satisfactory fitting shown in **Fig. 2C.** Here we show, by employing a more elaborate fitting procedure to fit the same experimental data, that we can extract more details on the crystal symmetry.

In the PGE (θ) measurement, the light source before the λ/4 plate is *p*-polarized, equivalent to $\beta=0^0$ in the general formula presented in the **Auxiliary Supplementary Materials**. The azimuthal angle ϕ in the theoretical calculation is related to the azimuthal angle ϕ' in experiment by: $\phi=\phi'-\psi$ (see **Fig. 1C** for the definitions of angles). The data in **Fig. 2C** were taken with $\phi'=90^0$, therefore $\phi=90^0-\psi$. The θ dependence of LPGE (*L2*) and CPGE (*C1*) are derived to be:

$$L2(\theta)=\frac{1}{4}sin\psi\{\cos(\psi)[(3+\cos(2\theta))\cos(\psi)\chi_{xxy}-\sin(2\theta)\,\chi_{xxz}]+(-\cos^2\psi+\cos^2\theta\sin^2\psi)\chi_{yxx}+(-\sin^2\psi+\cos^2\theta\cos^2\psi)\chi_{yyy}-sin(2\theta)\,cos\psi\chi_{yyz}\} \quad (14)$$

$$C1(\theta)=\sin(\theta)\left[\cos^2\psi\,\gamma_{xy}-\sin^2\psi\,\gamma_{yx}\right]-\cos(\theta)\cos(\psi)\,\gamma_{xz} \quad (15)$$

Please note that Supplementary equ. (14) and (15) can be simplified (by omitting details of symmetry tensors) as:

$$L2(\theta)=A'\sin 2\theta+B'\cos 2\theta+G \quad (14')$$

$$C1(\theta)=A\sin\theta+B\cos\theta \quad (15')$$

These are the equations used in the fitting shown in **Fig. 2C&D** in the main text, with various coefficients being:

$$A'=-\frac{1}{8}\sin(2\psi)\left(\chi_{xxz}+\chi_{yyz}\right)$$

$$B'=\frac{1}{8}\sin\psi\left[\cos^2\psi\left(2\chi_{xxy}+\chi_{yyy}\right)+(\sin^2\psi)\chi_{yxx}\right]$$

$$G=\frac{1}{8}\sin\psi\left[\chi_{yyy}(\cos(2\psi)-\sin^2\psi)-\chi_{yxx}(\cos(2\psi)+\cos^2\psi)+6\cos^2\psi\chi_{xxy}\right]$$

$$A=\cos^2\psi\,\gamma_{xy}-\sin^2\psi\,\gamma_{yx}$$

$$B=-\cos(\psi)\,\gamma_{xz}$$

The fitting parameters used in **Supplementary Figure 18** are 8 non-zero components of χ & γ tensors including: 2 non-zero components of χ tensor (χ_{xxz} and χ_{yyz}), and 2 non-zero components of γ tensor (γ_{xy} *and* γ_{yx}) for out-of-plane inversion asymmetry along z-axis; 3 non-zero components of χ tensor (χ_{xxy}, χ_{yxx} and χ_{yyy}), and 1 non-zero components of γ tensor (γ_{xz}) for in-plane inversion asymmetry along y-axis, as well as angle ψ between the current direction and the crystal a-axis as defined in **Fig. 1C**. Among all 9 parameters, the ones that matter are listed in **Supplementary Table 5**. The best fitting of *L2(θ)* (blue line in **Supplementary Figure 18)** shows that there are two non-zero elements χ_{yyy} and χ_{yxx}, confirming that the crystal symmetry is C_i with in-plane inversion symmetry breaking along the crystal *b*-

axis. The fitting of $C1(\theta)$ (orange line in **Supplementary Figure 18**) shows that, for the second-rank pseudo tensor γ_{ij}, the dominant term is γ_{yx}, indicating out-of-plane inversion symmetry breaking along z-direction. The fitting also yields near zero γ_{xz}, indicating negligible in-plane inversion symmetry breaking, which seems to contradict the result from the L2(θ) fitting. However, this apparent contradiction is incorrect. The fitting also yields ψ=87^0, meaning that the current direction chosen in our measurement is almost along the crystal *b*-axis (in-plane inversion symmetry breaking direction). In theory, the CPGE current is null when it is measured along the symmetry breaking axis [12]. Therefore it is expected that γ_{xz} is near zero. The appearance of in-plane symmetry breaking is subtler in the *C1* curve, but can be seen in the fact that the magnitude of *C1* looks systematically larger for $\theta > 0$ than it is for $\theta < 0$.

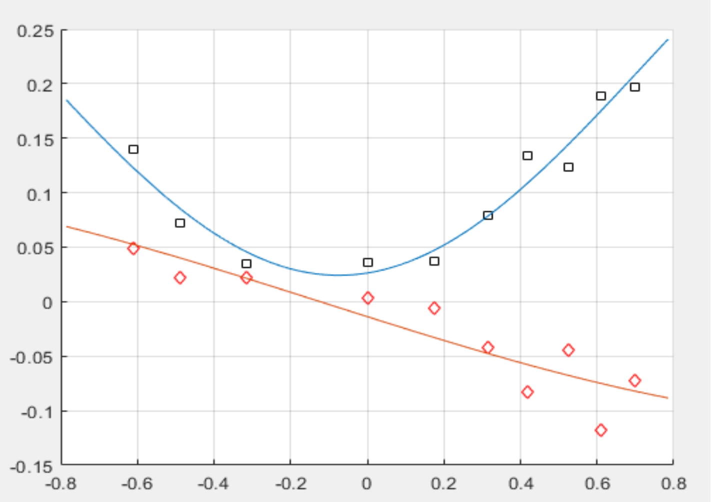

Supplementary Figure 18. Fitting of the extracted LPGE, $L2(\theta)$ (blue line) and CPGE, $C1(\theta)$ (orange line) parameters using Supplementary equ. (14) and equ. (15), respectively. The x-axis has the unit of radian. The open symbols are the experimental data that are also presented in **Fig. 2C**.

Supplementary Table 5. lists the five important parameters extracted using Supplementary equ. (14) and (15). The other four parameters do not affect the fitting results in noticeable way, and their values are not important.

χ tensor	χ_{yyy}	χ_{yxx}	γ tensor	γ_{yx}	γ_{xz}		ψ (rad/degree)
	-1.6	-1.5		0.11	<<0.1		1.52(87)

SM12. Two band calculation of the CPGE action spectrum

The orbital character of the conduction and valence band in 2D-PEPI is similar to that of 3D $MAPbI_3$. In particular, the conduction band is primarily spin-orbital split Pb p-orbital with total angular momentum $J = 1/2$, whereas the valence band is composed of Pb s-orbitals and I p-orbitals, with overall s=1/2 characteristic. The basis functions for the conduction and valence band electrons are therefore given by:

$$|J_{+1/2}\rangle = \frac{1}{\sqrt{3}}(|(X+iY)\downarrow\rangle + |Z\uparrow\rangle)$$

$$|J_{-1/2}\rangle = \frac{1}{\sqrt{3}}(|(X-iY)\uparrow\rangle - |Z\downarrow\rangle)$$

$$|S_{+1/2}\rangle = |S\uparrow\rangle$$

$$|S_{-1/2}\rangle = |S\downarrow\rangle$$

where X, Y, Z denote real-valued $L = 1$ spherical harmonic function p_x, p_y, p_z, respectively. As discussed in the main text, the experimental data indicates that the out-of-plane symmetry breaking is more substantial than the in-plane symmetry breaking. Accordingly, we consider a minimal $k \cdot p$ model with an out-of-plane symmetry-breaking Rashba term (along the z-direction). In the basis of $|J_{+1/2}\rangle$, $|J_{-1/2}\rangle$, $|S_{+1/2}\rangle$, $|S_{-1/2}\rangle$, the Hamiltonian is given by:

$$H = \begin{pmatrix} (k_x^2+k_y^2)t_p+\epsilon_0 & (ik_x+k_y)\alpha_c & 0 & -(ik_x+k_y)\xi \\ (k_y-ik_x)\alpha_c & (k_x^2+k_y^2)t_p+\epsilon_0 & -(ik_x-k_y)\xi & 0 \\ 0 & -(-ik_x-k_y)\xi & (-k_x^2-k_y^2)t_s & (ik_x+k_y)\alpha_v \\ -(k_y-ik_x)\xi & 0 & (k_y-ik_x)\alpha_v & (-k_x^2-k_y^2)t_s \end{pmatrix},$$

where $t_{p(s)}$ is the intra-orbital hopping between $p(s)$ bands, $\alpha_{c(v)}$ is the Rashba coefficient for conduction(valence) bands respectively, and ξ is the interband hybridization which enables electron-hole couplings. Note that k is taken to be dimensionless (scaled by inverse lattice constant a^{-1}), and $\alpha_{c(v)}$ is given in units of energy.

The general expression for the circular photogalvanic effect at zero temperature is given as [13]:

$$\gamma_{ij} = \tau\frac{2\pi e^3}{\hbar^2}\int d\boldsymbol{k}\,\epsilon_{j\ell m}\sum_{cv}\left|M_{cv}^{\ell m}(\boldsymbol{k})\right|^2\delta(E_c(\boldsymbol{k})-E_v(\boldsymbol{k})-\hbar\omega)\left(\frac{dE_c}{dk_i}-\frac{dE_v}{dk_i}\right),$$

where τ is the momentum relaxation time, $\epsilon_{j\ell m}$ is the antisymmetric tensor, $M_{cv}^{\ell m} = \langle\psi_c|(\hat{r}^\ell + i\hat{r}^m|\psi_v\rangle$. $\hat{r}^\ell$ is the dipole operator, which for extended dimensions is related to the velocity operator: $\langle\psi_c|\hat{r}^\ell|\psi_v\rangle = i\left\langle\psi_c\left|\frac{dH}{dk_\ell}\right|\psi_v\right\rangle/(E_c-E_v)$. For the 2D model, we cannot define a velocity matrix with z component. However, the conduction bands include a Z-orbital component which leads to nonzero dipole matrix in z direction,

$$r_z = \begin{pmatrix} 0 & 0 & -z & 0 \\ 0 & 0 & 0 & z \\ -z^* & 0 & 0 & 0 \\ 0 & z^* & 0 & 0 \end{pmatrix},$$

where z parameterizes the oscillator strength.

Expanding in the small parameter ξ/ϵ_0, we obtain the following closed form expression for the photogalvanic tensor for current along the x-direction upon absorption of incident light circularly polarized along the y-direction:

$$\gamma_{xy} = \frac{2\xi\mathrm{Re}(z)}{(\hbar\omega)^2 t}\times\begin{cases} 2\sqrt{t(E_R+\hbar\omega-\epsilon_0)}(4E_R+\hbar\omega-2\epsilon_0) & \text{for } \epsilon_0-E_R\le\hbar\omega\le\epsilon_0 \\ \alpha(4E_R+3\hbar\omega-4\epsilon_0) & \text{for } \hbar\omega>\epsilon_0 \end{cases}$$

where $t = t_s + t_p$, $\alpha = \alpha_c + \alpha_v$. This response exhibits a peak at $\hbar\omega = E_R = (\alpha_c + \alpha_v)^2/4(t_s + t_p)$. Note that this expression is valid for any *sign combination* of Rashba splitting for valence and conduction band. This expression can be formulated in terms of effective mass model of band edges by identifying $t_{c,v} = \hbar^2/(2a^2 m^*_{c,v})$, where a is the lattice constant. Then $E_R = E_d - (m^*_c + m^*_v)\,(\alpha_c + \alpha_v)^2/(2\hbar^2)$.

The addition of broadening substantially smears out the tail of the response (the analytical expressions are cumbersome and not presented here). The numerical expression for γ_{ij} including a broadening energy η is:

$$\gamma_{ij} = \tau \frac{2e^3}{\hbar^2} \int d\boldsymbol{k}\, \epsilon_{j\ell m} \sum_{cv} \left| M_{cv}^{\ell m}(\boldsymbol{k}) \right|^2 \frac{\eta}{\eta^2 + (E_c(\boldsymbol{k}) - E_v(\boldsymbol{k}) - \hbar\omega)^2} \left(\frac{dE_c}{dk_i} - \frac{dE_v}{dk_i} \right).$$

The large impact of smearing can be understood as a consequence of the large density of states at the conduction/valence band edges. The shift of the band extrema away from $k = 0$ leads to a density of states that diverges as $E^{-1/2}$ as the energy approaches the band edge. Significant spectral weight is therefore available to be shifted from above the band edge to below. Numerically, we find that a qualitatively similar spectrum is obtained over a range of Rashba energies E_R and smearing energies (or broadening) η when both are in the range of 10's of meV. The choice of broadening parameter is consistent with other references [14]. This provides a limit on the precision with which the model determines the value of Rashba splitting. The fitting result is shown in part in **Fig. 2B**, with **Supplementary Figure 19 (A)** shows the range of parameters for E_R and η in unit of meV. manuscript.

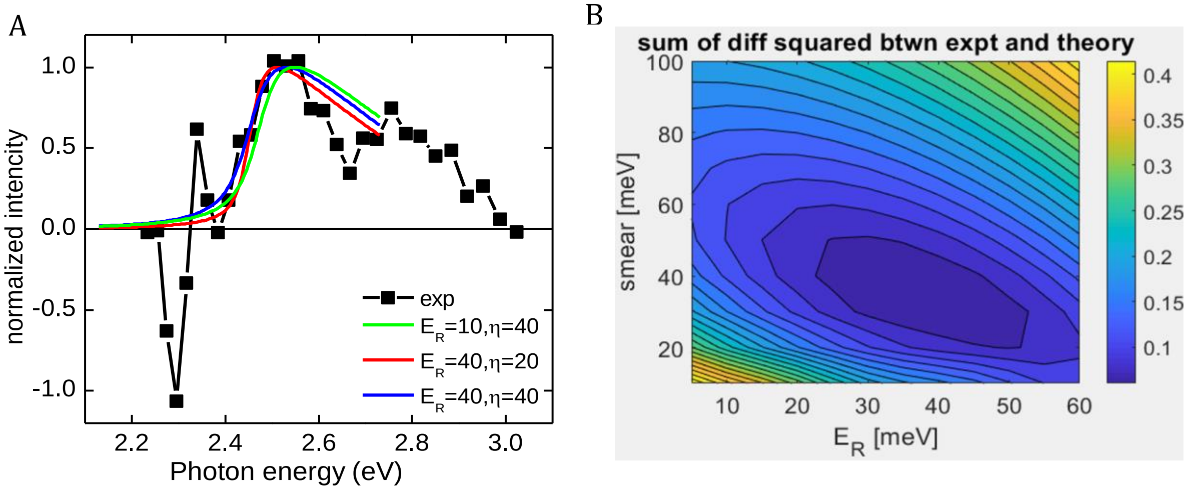

Supplementary Figure 19. (**A**). Comparison of experimental CPGE action spectrum (also shown in **Fig. 2B** in the main text) with calculation using different Rashba energies (E_R) and broadening parameters (η). (**B**). Sum of difference squared between computed and measured CPGE values, as a function of Rashba energy E_R and broadening parameter η. The experimental values used for comparison are limited to the energy range 2.38 eV to 2.55 eV.

More accurate estimate may be achieved when fitting with low temperature data which is under way. **Supplementary Figure 19 (B)** shows a comparison of the model fit to experimental data as a function of Rashba splitting energy E_R and broadening parameter η. We compute the sum of the squares of the difference between the (normalized) measured and computed values of the CPGE intensity. Based on this

result we obtain Rashba splitting (E_R) of 35±10 meV and broadening parameter (η) of 30±10 meV as given in the

Supplementary References

1. Lédée, F. *et al*. Fast growth of monocrystalline thin films of 2D layered hybrid perovskite. *CrystEngComm* **19**, 2598-2602 (2017).
2. Osterhoudt, G. B. *et al*. Colossal mid-infrared bulk photovoltaic effect in a type-I Weyl semimetal. *Nature Materials* **18**, 471–475 (2019).
3. The full description of the procedures used in this paper requires the identification of certain commercial products. The inclusion of such information should in no way be construed as indicating that such products are endorsed by NIST or are recommended by NIST or that they are necessarily the best software for the purposes described.
4. Niesner, D. *et al.* Structural fluctuations cause spin-split bands in $(CH_3NH_3)PbI_3$: Experimental evidence from circular photogalvanic effect. *PNAS*, **115**, no. 38, 9509–9514(2018).
5. Zhai, Y. *et al.*, Giant Rashba splitting in 2D organic-inorganic halide perovskites measured by transient spectroscopies, *Sci. Adv.* **3**: e1700704 (2017).
6. Blancon, J.-C. *et al*. Extremely efficient internal exciton dissociation through edge states in layered 2D perovskites. *Science* **355**, 1288–1292 (2017).
7. Fieramosca, A. *et al.* Out-of-Plane Excitons in 2D Single-Crystal Perovskites. *ACS Photonics* **5**, 4179−4185(2018).
8. Calabrese, J. *et al.* Preparation and Characterization of Layered Lead Halide Compounds. *J. Am. Chem. Soc*. **113**, (2328-2330)1991.
9. Mao, L. et al., Hybrid Dion−Jacobson 2D Lead Iodide Perovskites, J. Am. Chem. Soc. 2018, 140, 3775−3783.
10. M. Sender *et al.*, Optical phonons in methylammonium lead halide perovskites and implications for charge transport, Materials Horizons 3, 613-620 (2016).
11. Vikhnin, V.S., Eglitis, R.I., Kapphan, S.E., Borstel, G., Kotomin, E.A., Polaronic-type excitons in ferroelectric oxides: Microscopic calculations and experimental manifestation. *PHYSICAL REVIEW B*, **65**, 104304 (2002).
12. Ganichev,S.D. *et al.*, Removal of spin degeneracy in p-SiGe quantum wells demonstrated by spin photocurrents, PHYSICAL REVIEW B **66**, 075328 (2002).
13. Aversa, C., J. E. Sipe, J.E., Nonlinear optical susceptibilities of semiconductors: Results with a length-gauge analysis. *Phys. Rev. B* **52** 14636 (1995).
14. Neutzner, S. *et al.* Exciton-polaron spectral structures in two-dimensional hybrid lead-halide perovskites. *PHYSICAL REVIEW MATERIALS* **2**, 064605 (2018).
